# Genetic manipulation of iron biomineralization enhances MR relaxivity in a ferritin-M6A chimeric complex

**DOI:** 10.1038/srep26550

**Published:** 2016-05-23

**Authors:** Marina Radoul, Limor Lewin, Batya Cohen, Roni Oren, Stanislav Popov, Geula Davidov, Moriel H. Vandsburger, Alon Harmelin, Ronit Bitton, Jean-Marc Greneche, Michal Neeman, Raz Zarivach

**Affiliations:** 1 Department of Biological Regulation, Weizmann Institute of Science, Rehovot 76100 Israel; 2 Department of Life Sciences and the National Institute for Biotechnology in the Negev, Ben-Gurion University of the Negev, POB 653, Beer-Sheva 84105, Israel; 3 Department of Veterinary Resources, Weizmann Institute of Science, Rehovot 76100 Israel; 4 Department of Chemical Engineering and Ilse Katz Institute for Nanoscale Science and Technology Ben-Gurion University of the Negev, 84105 Beer-Sheva, Israel; 5 Institut des Molécules et Matériaux du Mans (IMMM), UMR CNRS 6283 Université du Maine, Avenue Olivier Messiaen, 72085 Le Mans Cedex France

## Abstract

Ferritin has gained significant attention as a potential reporter gene for *in vivo* imaging by magnetic resonance imaging (MRI). However, due to the ferritin ferrihydrite core, the relaxivity and sensitivity for detection of native ferritin is relatively low. We report here on a novel chimeric magneto-ferritin reporter gene – ferritin-M6A – in which the magnetite binding peptide from the magnetotactic bacteria magnetosome-associated Mms6 protein was fused to the C-terminal of murine h-ferritin. Biophysical experiments showed that purified ferritin-M6A assembled into a stable protein cage with the M6A protruding into the cage core, enabling magnetite biomineralisation. Ferritin-M6A-expressing C6-glioma cells showed enhanced (per iron) r_2_ relaxivity. MRI *in vivo* studies of ferritin-M6A-expressing tumour xenografts showed enhanced R_2_ relaxation rate in the central hypoxic region of the tumours. Such enhanced relaxivity would increase the sensitivity of ferritin as a reporter gene for non-invasive *in vivo* MRI-monitoring of cell delivery and differentiation in cellular or gene-based therapies.

Ferritin, the highly conserved intracellular iron storage protein, is one of the most extensively studied magnetic resonance imaging (MRI) reporter genes^1^,^2^,^3^,^4^,^5^. The interest in ferritin stems from its unique biological, biophysical and biochemical properties. Ferritin iron is stored as ferrihydrite resulting in magnetic field-dependent enhancement of R_2_ relaxation. This enhancement can be used for non-invasive quantitative mapping of endogenous physiological ferritin iron stores *in vivo* by MRI^6^. As an iron oxide-based contrast material, ferritin relaxivity is low relative to either superparamagnetic iron oxides or magnetite (8000–63000 μB), since most of the iron magnetic spin moments in the ferrihydrite core are aligned antiferromagnetically and balance each other, whereas only uncompensated spins on the core surface sum up to the net magnetic moment of ~300 μB^7^,^8^,^9^.

Ferritin is a spherical, oligomeric, hollow, iron-storage protein complex composed of 24 subunits. In vertebrates, ferritin is a hetero-oligomeric complex composed of two homologous chains, light (l-ferritin) and heavy (h-ferritin). Only the h-ferritin chain exhibits ferroxidase activity that converts iron from the ferrous (Fe^2+^) to the ferric (Fe^3+^) state. The ferritin cage is able to store up to ~4500 iron atoms as ferrihydrite^10^. Under non-physiological conditions, ferrihydrite can be converted into magnetite (Fe_3_O_4_) and then into maghemite (γ-Fe_2_O_3_) within the ferritin core through oxygen reduction and heating (magnetite), followed by oxidation (maghemite), thereby creating a magnetoferritin^10^,^11^. Insight to the impact of altering the iron core of ferritin can be obtained from Clavijo Jordan *et al*.^12^ which report on the chemical synthesis of magneto-ferritin resulting in a change of per iron spin-echo derived *r*_*2*_ relaxivity of 4 mM^−1^s^−1^ for native ferritin to 130 mM^−1^s^−1^ for magneto-ferritin.

Biological synthesis of magnetite can be found in a number of organisms including magnetotactic bacteria, which deposit magnetite under microaerophilic (hypoxic to anaerobic) conditions^13^,^14^,^15^. The biosynthesis takes place in the magnetosome, a specialised lipid vesicle, which includes unique integral-membrane magnetosome-associated proteins (MAPs) that control the nucleation and growth of magnetite crystals^16^.

Amongst MAPs, Mms6 was demonstrated *in vitro* to interact directly with the magnetite surface^17^,^18^. The active component of Mms6 has been identified as the hydrophilic C-terminal 12 amino acids tail (M6A) that is sufficient to affect magnetite size and shape^18^. *In vivo* studies on magnetotactic bacteria have revealed magnetite crystal deformation caused by deletion of Mms6^19^. Recent studies showed that Mms6 binds iron in a two-phase way: high affinity stoichiometric and low affinity with high capacity^20^. These studies revealed a self-assembled micelle-like formation *in vitro* due to the amphipathic nature of Mms6 protein. As a way to harness MAPs as reporter genes for MRI, several magnetotactic bacteria proteins, including MagA and Mms6, were expressed in eukaryotic cells^21^,^22^,^23^,^24^.

We report here on a novel platform for utilising the ferritin cavity for genetic manipulation of biomineralisation pathways in mammalian cells. The ability of ferritin to concentrate endogenous iron was combined with that of Mms6 to affect the magnetite crystal formation through the interaction of M6A with iron oxide, so as to genetically induce the formation of magneto-ferritin in living cells (Supplementary Fig. 1). X-ray crystallography, small angle X-ray scattering (SAXS) and transmission electron microscopy (TEM) confirmed that such an addition did not interfere with the assembly of ferritin, while enhanced per iron relaxivity was demonstrated by MRI.

## Results

### *In vitro* analysis of ferritin-M6A

**Figure 1:**
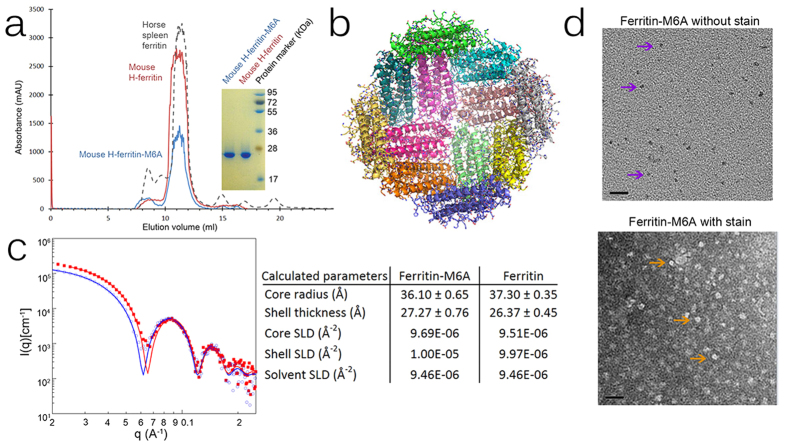
Structural analysis of wild type mouse h-ferritin and chimeric mouse h-ferritin-M6A. (**a**) Size-exclusion chromatography (SEC) of recombinant ferritin-M6A and ferritin (left), and SDS-PAGE analysis of the corresponding SEC peaks (right). (**b**) The determined ferritin-M6A structure; each colour represents a monomeric chain. (**c**) SAXS data of purified ferritin-M6A (blue circles) and ferritin (red square) analysed with a sphere core-shell model (red and blue lines) yielding the model parameters (right Table). (**d**) TEM images of unstained (top) and negatively stained (bottom) recombinant iron-loaded ferritin-M6A. Black bars indicate 20 nm length; arrows indicate representative ferritin complexes.

The ability to induce biomineralisation of magnetite within the ferritin core was tested using a chimeric ferritin-M6A construct in which the M6A peptide from Mms6 was added to the h-ferritin C-terminal. We first tested the structural stability and functional properties of ferritin-M6A *in vitro*. Ferritin and ferritin-M6A proteins were expressed to the same level in *E. coli* Rosetta(DE3)pLysS strain cells and were purified using Ni-NTA affinity and size-exclusion chromatography. Size-exclusion chromatography indicated that both proteins were highly soluble and stable with a nano-cage packing, as they both eluted at a volume corresponding to an assembly of 24 monomers with a molecular weight of ~440 kDa, which is similar to horse spleen ferritin (Fig. 1a).

To gain better understanding of the ferritin-M6A assembly we conducted X-ray crystallography studies. Ferritin-M6A was crystallised and diffracted to 2.25 Å resolution (Supplementary Table 1). As can be seen (Fig. 1b), the ferritin-M6A structure adopts the ferritin fold with a four-helix bundle and a C-terminal helix located at the four-fold symmetry of the well-organised protein shell. The structure contains the metal ion channels and the ferroxidase centres. Mouse ferritin-M6A is highly similar to human h-ferritin (PDB 3AJO)^25^, both in sequence and in structure, with RMSD of 0.3 Å over Cα atoms and with only 10 residues differing out of a 180-residue sequence (see detailed analysis in Supplementary Fig. 2).

As the N- and C-terminals of ferritin are flexible, no electron density was found for the M6A peptide (residues 184–195). Yet, to confirm that the Mms6 peptide is present at the C-terminal and to test the packing of ferritin-M6A in solution, we performed SAXS measurements on purified ferritin and ferritin-M6A. Both scattering curves were fit to a spherical core-shell model. The SAXS analysis indicates that both proteins oligomerise in solution into a symmetric homo-oligomeric sphere with core and shell dimensions the same size as was seen in the crystal structure (Fig. 1c). The best-fit parameters indicate that ferritin-M6A exhibited higher core density than ferritin, although both proteins were purified and stored in identical buffers. This difference may be attributed to the existence of 24 flexible M6A peptides inside the ferritin-M6A core. However, the differences are small and a definite conclusion based solely on the SAXS data cannot be made. To further validate the presence of the M6A in the ferritin we performed MALDI-TOF analysis of purified h-ferritin-M6A. The measured ferritin-M6A size (25.843 kDa) is within the calculated size range (25.868 kDa) (Supplementary Fig. 3).

To test whether M6A peptides interfere with the ability of ferritin-M6A to absorb and store iron, we examined ferritin-M6A iron incorporation. Ferritin particles are known to consist of a protein shell of ~12 nm in diameter and an iron core of ~6 nm in diameter. Using TEM, stained ferritin-M6A particles were discerned as approximately 6 nm black particles (Fig. 1d). Negatively stained ferritin-M6A particles were discerned as ~12 nm white rings (Fig. 1d), as were seen earlier for native h-ferritins (Supplementary Fig. 4) and for other ferritin particles^26^. The TEM results indicate that ferritin-M6A is able to oligomerise in solution and absorb iron while in the cage form.

In addition to the TEM analysis, ferritin-M6A activity was confirmed by a detectable iron uptake using the protein in its crystal form. Ferritin-M6A crystals that were soaked with ammonium ferrous sulphate changed their colour from transparent to red, which corresponds to the mixture of iron with oxo-hydroxides in the ferritin core (Supplementary Fig. 5). Attempts to measure these crystals by X-ray diffraction failed due to crystal cracking and resolution loss.

### MRI detection of enhanced iron uptake and relaxivity in ferritin-M6A expressing cells

**Figure 2:**
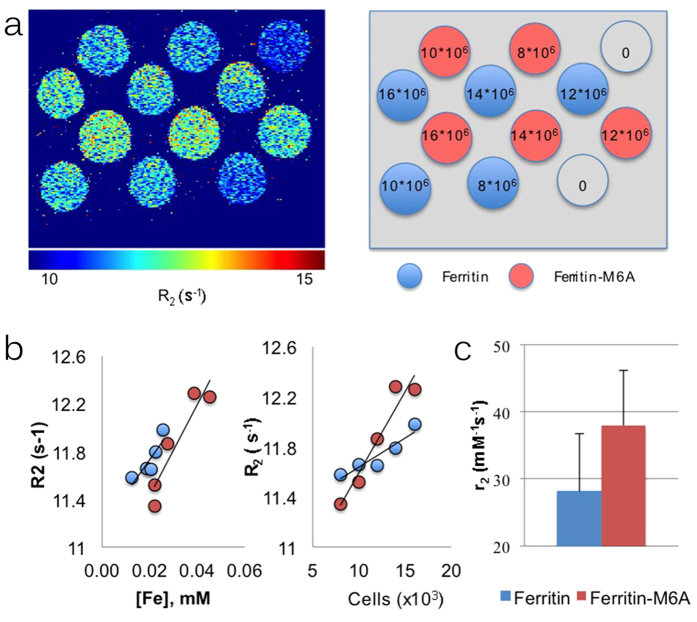
*In vitro* MRI measurements of iron-induced relaxivity in rat C6 glioma cells overexpressing either HA-tagged mouse h-ferritin or HA-tagged ferritin-M6A. (**a** left) R_2_ relaxation map of cell phantom. (**a** right) Schematic representation of R_2_ map (as presented at the left) shows the cells transfected either with HA-HFn (blue circles) or with HA-HFn-M6A (red circles). The numbers indicate the number of cells. Note the different dependence of R_2_ on iron concentration (**b** left) and on cell number (**b** right). (**c**) Specific relaxivity (**r**_**2**_; mM^−1^s^−1^; ±SE) was calculated from the linear change in R_2_ as a function of intracellular iron content.

In order to test the relaxivity properties of ferritin-M6A as an MRI reporter gene, rat glioma C6 cells were stably transfected with either haemagglutinin-tagged (HA) mouse h-ferritin (HA-HFn) or HA-tagged ferritin-M6A (HA-HFn-M6A) and selected with puromycin (2.5 μg mL^−1^). The cells were initially used to measure R_2_ relaxation rates (s^−1^) for derivation of the specific **r**_**2**_ relaxivity (mM^−1^s^−1^). Different numbers of cells were imaged in the test tubes on top of a cooled 1% low melting-point agarose. R_2_ relaxation rates were elevated in C6-HFn-M6A cells compared to those of C6-HFn. The iron content of the cells was determined after the MRI measurements using ICP-MS, and used for calculation of the **r**_**2**_ relaxivity. Iron content per cell was significantly elevated in the C6-HFn-M6A cells (p = 0.00023). Notably, **r**_**2**_ relaxivity (per iron) in C6-HFn-M6A cells was significantly elevated relative to C6-ferritin cells, consistent with a change in the ferritin core mineral (Fig. 2).

Subsequently, the cells were used for *in vivo* analysis of tumour xenograft in CD-1 nude mice. Rat glioma C6 cells, cells overexpressing either HA-HFn or HA-HFn-M6A, were inoculated subcutaneously. Histogram analysis of R_2_ maps derived from the tumours at three weeks after inoculation revealed enhanced relaxation for both HA-HFn- and HA-HFn-M6A-expressing tumours. Elevated R_2_ values could be detected for HA-HFn-M6A-expressing tumours relative to HA-HFn overexpressing tumours (Supplementary Fig. 6).

### Mössbauer analysis

**Figure 3:**
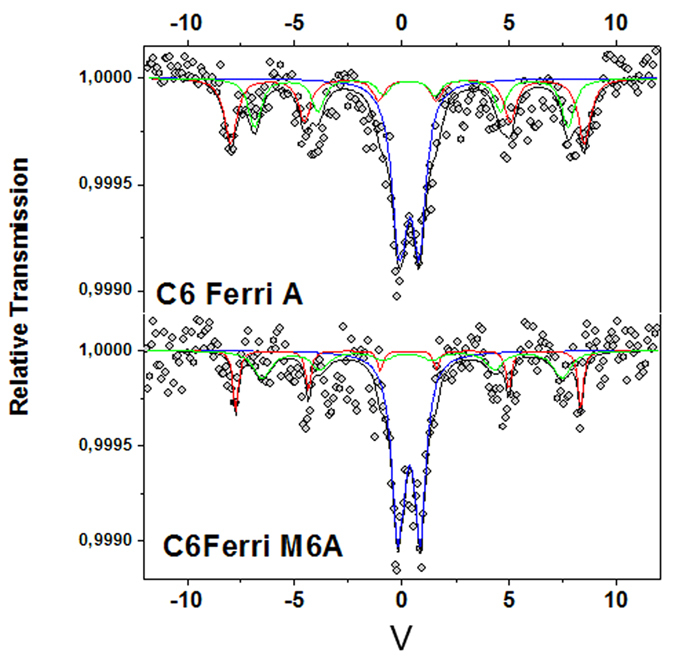
Mössbauer spectra recorded at 77 K on the C6 ferritin (C6FerriA) and Ferritin-M6A (C6FerriM6A) expressing tumours. Blue distiguishes the quadrupolar component assigned to Fe species in superparamagnetic nanoparticles and the red and green magnetic components assigned to larger nanoparticles and slowly relaxing intermediate size nanoparticles.

To test for the differences of the mineral cores *in vivo* we performed Mössbauer analysis on ferritin expressing tumours (Fig. 3). One can distinguish on the 77 K Mössbauer spectra magnetic sextets and a central quadrupolar doublet. The poor statistics does not allow an easy description of the hyperfine structure (note the very low relative transmission); in addition, such a feature prevents from performing Mössbauer spectra versus temperature and/or external magnetic field. Nevertheless, the isomer shift of the different components and the mean value are estimated at values ranged from 0.45 and 0.55 mm/s at 77 K (quoted relative to that of metallic Fe at 300 K). They are unambiguously consistent with the presence of different HS Fe^3+^ species, excluding thus the presence of pure magnetite. The quadruplar component can be a priori attributed to non-interacting nanoparticles the diameter of which is below about 6–8 nm, the size corresponding to ferritin cores. Indeed, such size favours the occurrence of superparamagnetic fluctuations resulting from the dynamics of magnetic moments. It is important to emphasize that the relative proportions of these Fe species which are estimated from the corresponding absorption areas, assuming the same values of their recoilless Lamb-Mössbauer factors, are slightly different between the two samples. The quadrupolar component appears significantly larger in the case of the M6A sample allowing us to conclude that it contains smaller nanoparticles. The magnetic components are rather due to larger interacting nanoparticles. One distinguishes two different magnetic Fe^3+^ species: the external magnetic component (red line) could be attributed to well blocked nanoparticles, i.e. large nanoparticles while the second component is *a priori* assigned to slowly relaxing nanoparticles, i.e. with intermediate size. But at this stage, different scenarios can be considered and it remains difficult to model the hyperfine structure from experimental data coming from the measurements obtained at one temperature.

### Ferritin-M6A reveals hypoxic regions in C6 glioma tumours

**Figure 4:**
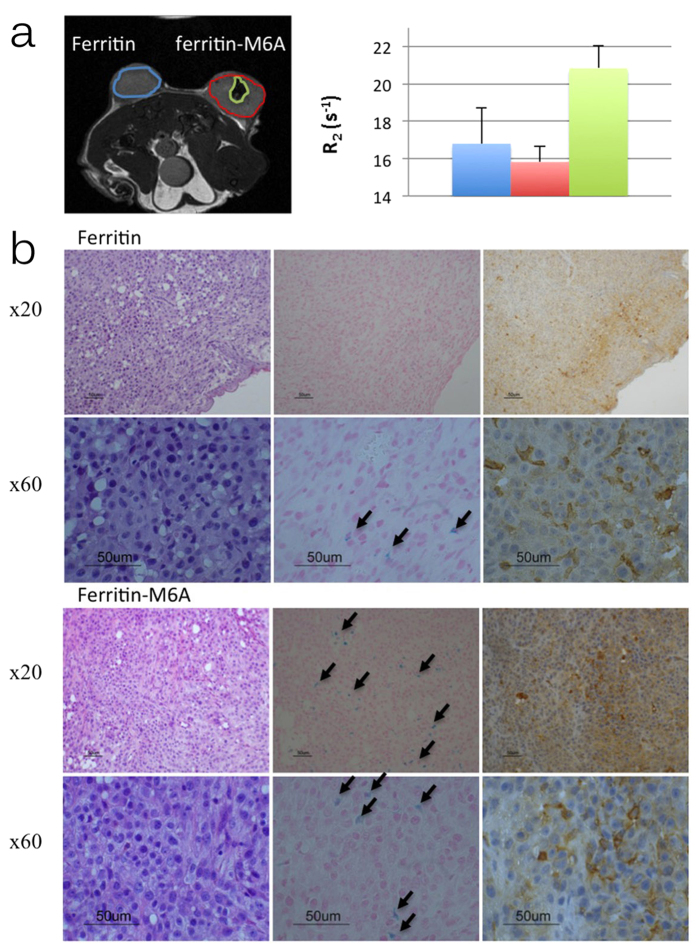
Ferritin M6A expression results in enhanced MRI contrast in hypoxic regions of tumour xenografts. (**a** left) *In vivo* R_2_ map of the axial slice through the centre of both the C6-HA-HFn (left) and C6-HA-HFn-M6A (right) tumours. (**a** right) Bar graph represents mean R_2_ values (±SD) measured at the C6-HA-HFn tumours (blue), C6-HA-HFn-M6A tumours (red) and the central hypoxic region of C6-HA-HFn-M6A tumours (green). A significant increase in R_2_ relaxation rate was detected in the central hypoxic region of C6-HA-HFn-M6A (n = 3; p < 0.05). (**b**) Histological analysis of C6-HA-HFn (upper panel) and C6-HA-HFn-M6A (lower panel) tumours stained with (left) Hematoxylin-Eosin (HE) to examine cell structure; (centre) Prussian blue to evaluate iron accumulation in cells (arrows, Prussian Blue stained cells); (right) Immunihistostaining with Hypoxyprobe (pimonidazole) counterstained with haemotoxylin to confirm hypoxic regions.

The spatial distribution of high relaxivity HA-HFn-M6A cells was evaluated using mice in which C6 glioma cells overexpressing either HA-HFn or HA-HFn-M6A were inoculated subcutaneously above the gluteal muscle on both sides of the mouse. Three weeks after inoculation, when the tumours reached approximately 0.8 cm in diameter, MRI measurements were acquired from a single axial slice through the centre of both tumours. C6-HA-HFn-M6A tumours demonstrated elevated R_2_ relaxation in the central hypoxic region of the tumour (Fig. 4a). Taking into consideration the hypoxic requirements for magnetite formation in magnetotactic bacteria, we postulated that a hypoxic region in the centre of the tumour might have a preferable environment for magnetite formation in C6-HA-HFn-M6A tumours. Magnetite can further oxidize into magnetic maghemite, an oxidation product of magnetite, thus explain both the elevated R_2_ values detected by MRI and the existence of Fe^3+^ species as was seen by the Mössbauer analysis. Analysis of the effects of hypoxia on iron uptake in C6-HA-HFn and C6-HA-HFn-M6A cells was indeed confirmed *in vitro* (Supplementary Fig. 7).

### Intracellular localisation of ferritin-M6A in hypoxic tumour regions

To detect hypoxic regions in HA-HFn and HA-HFn-M6A tumours, mice were injected with the hypoxia marker pimonidazole (60 mg kg^−1^; n = 2) one hour before sacrificing. The mice were sacrificed and the tumours were harvested for histological examination. The slices of sectioned tumours were stained with Hematoxylin-Eosin, Prussian blue and with pimonidazole, immunohistochemical staining of hypoxia (Hypoxyprobe; Fig. 4b right). Prussian blue staining was performed in order to detect intracellular iron in tumour cells. C6-HA-HFn-M6A tumours revealed significantly higher iron content, especially in the central parts of the tumours, while C6-HA-HFn tumours showed only a small amount of iron, which was mainly concentrated in the peripheral regions of the tumour (Fig. 4b centre). The cells showed nuclear pleomorphism with foci of tumour necrosis and vascular proliferation. In both cases, tumours showed multifocal areas of iron depositions in their cytoplasms, however iron distribution was much higher in all C6-HA-HFn-M6A tumours. Positive cells showed intra-cytoplasmic blue stains with variable deposit patterns. In contrast to C6-HA-HFn tumour cells, C6-HA-HFn-M6A tumours exhibited lipid vacuoles loaded with iron. Regions with high iron content, according to Prussian blue staining found in HA-HFn-M6A tumours, were confirmed to be hypoxic by immunohistochemistry (Fig. 4b right).

## Discussion

With the advances in gene- and cell-based therapies for regenerative medicine and for cancer therapy, non-invasive imaging of the location and fate of cells becomes critical. Reporter genes offer the prospect for utilising regulatory elements in the genome for non-invasive detection of the regulation of gene expression. This capability can potentially be used for tracking not only cell location but also its proliferation and differentiation. One of the most widely used reporter genes for MRI is the iron binding protein ferritin, which we and others introduced due to its endogenous MRI contrast^1^,^2^,^4^. However, as an iron-based nano-particle, ferritin is a weak MRI contrast agent due to the structure of the ferrihydrite mineral core. Attempts to genetically improve the contrast generated by ferritin include a recent introduction of mitochondrial ferritin modified so as to localise to the cell cytoplasm, resulting in increased iron load and higher relaxivity^27^.

In the study presented here, we aimed to genetically alter, for the first time, the biomineralisation process within the core of ferritin *in vivo*, so as to induce deposition of magnetic minerals, magnetite or maghemite rather than non-magnetic ferrihydrite. To induce a change in the iron mineralisation, a peptide involved in the magnetite mineralisation in magnetotactic bacteria was fused to the C-terminus of h-ferritin, thus allowing its protrusion into the cavity of the assembled ferritin complex. The chimeric HFn-M6A generated particles with a structure similar to native ferritin and were able to load and store iron.

*In vivo* MRI relaxation measurements have shown elevated relaxivity (per iron) in HFn-M6A tumours when compared to HFn tumours, which was further confirmed by showing a change in general iron mineral by Mössbauer spectrometry. Additionally, HFn-M6A cells have shown higher iron content then HFn expressing cells which can be the result of decrease iron release from the ferritin due to ferritin mineral core stabilization or due to increased iron uptake by HFn-M6A expressing cells. Moreover, hypoxia appeared to be a significant factor in relaxivity, which is higher in hypoxic regions in HFn-M6A tumours relative to nonhypoxic regions although both have higher iron content then HFn. This finding is consistent with the hypoxic requirement of magnetotactic bacteria for magnetite biomineralisation in the magnetosomes prior to further conversion into other magnetic nanoparticles such as maghemite.

Altogether, the increase of iron uptake by cells together with higher relaxivity per iron is the first step towards genetic control of the mineralisation process within the cavity of this widely bio-compatible storage protein in a manner that would affect its magnetic properties and its relaxivity. The ability to control iron biomineralisation suggests also the possibility for genetic control of the formation of additional minerals for therapeutic and diagnostic purposes. In particular, this strategy could aid in formation of biominerals sequestered in a biocompatible envelope, under physiological conditions, using the mechanisms harnessed by biological organisms for biomineralisation. Since human ferritin is highly conserved both in structure and in sequence, addition of a small peptide in its core is expected to yield similar behaviour. Thus, modified human ferritin may act as a genetic marker for future MRI studies.

## Materials and Methods

### Expression of ferritin and ferritin-M6A

*E. coli* Rosetta(DE3)pLysS cells harbouring plasmid pET28a-ferritin and pET28a-ferritin-M6A were grown in M9 medium containing kanamycin (50 mg mL^−1^) and chloramphenicol (30 mg mL^−1^) at 37 °C. Growth continued to OD of 0.6 at 600 nm, whereupon the expressions of plasmid-encoded proteins were induced by 0.5 mM IPTG at 37 °C for 4 h.

### Ferritin and ferritin-M6A purification

Ferritin- and ferritin-M6A-expressing cells were suspended in a binding buffer (20 mM Tris pH 8, 200 mM KCl, 10% glycerol, 2 mM TCEP and 20 mM imidazole) and incubated with DNase I (1 mg mL^−1^) and EDTA-free protease-inhibitor cocktail (100 mM phenylmethylsulphonyl fluoride (PMSF), 1.2 mg mL^−1^ leupeptin and 1 mM pepstatin A) in a ratio of 1:1000 with binding buffer. The cells were then disrupted by two cycles in a French press cell at 298 K followed by centrifugation at 17,210 g for 1 h at 298 K. The soluble fractions were applied onto a pre-equilibrated homemade gravity Ni-NTA column at room temperature containing Ni-NTA His-Bind resin. Proteins were washed with the Washing Buffer (20 mM Tris-HCl pH 8, 1 M KCl, 40 mM imidazole, 10% glycerol and 2 mM TCEP) and were eluted with Elution Buffer (20 mM Tris pH 8, 200 mM KCl, 10% glycerol, 2 mM TCEP, 50 mM EDTA) after a minimum of 5 h of incubation with the resin. The eluted proteins were mixed with thrombin from bovine plasma (T7513-500UN), 10 units per 1 mg proteins, to remove the histidine tag, and then applied onto a size-exclusion column (HiLoad 26/60 Superdex 200, GE Healthcare Biosciences) pre-equilibrated with Equilibration Buffer (20 mM Tris-HCl pH 8, 200 mM KCl, 10% glycerol and 2 mM TCEP). Purified ferritin and ferritin-M6A were depleted from iron and nickel traces by dialysis against the equilibration buffer containing thioglycolic acid (0.15 M pH 8), followed by dialysis against the same buffer without thioglycolic acid. Purified ferritin and ferritin-M6A were concentrated to 20 mg mL^−1^. Samples’ purities at this stage were analysed by SDS-PAGE and proteins’ identities were confirmed by tandem mass spectroscopy.

### Crystallisation and structure determination

Purified ferritin-M6A was crystallised using the sitting-drop vapour-diffusion method at 293 K. 0.5 mL ferritin-M6A (20 mg mL^−1^) and 0.5 mL reservoir solution (0.1 M Na-cacodylate pH 6.5, 0.2 M magnesium acetate and 30% MPD) were mixed to form the drop.

Crystals were harvested and flash-cooled in liquid nitrogen without addition of cryoprotecting solution. Diffraction data were collected on beamline ID14-4 of the ESRF (Grenoble, France) equipped with an ADSC Q4 CCD detector. Data collection was performed at 100 K. A total of 530 frames were collected with an oscillation range of 0.25° and an exposure time of 0.1 s per image. The crystal-to-detector distance was 290.96 mm. Data were reduced and scaled using the HKL2000 suite^28^. Ferritin-M6A phases were obtained using Phaser molecular replacement^29^ and PDB code: 1IES^30^ as a template. The final model was built by Coot^31^ and refined in REFMAC^32^. For Rfree calculation, 5% of the data were excluded. Structural figures were prepared with PyMOL^33^.

RMS calculations were performed with SwissPDB viewer^34^ over all Cα atoms using the domain alternate fit to align structures on the basis of the conserved domain.

### Small angle X-ray scattering (SAXS) measurements and model fitting

Prior to performing the SAXS experiments all protein samples were subjected to SEC purification to eliminate products of complex formation or aggregation. Ferritin and ferritin-M6A samples were diluted each with 20 mM Tris pH 8, 200 mM NaCl, 5 mM β -mercaptoethanol and 10% glycerol. SAXS measurements were performed at the French national synchrotron facility, SOLEIL, on the SWING beamline. The incident beam energy was 12 keV. The sample-to-detector (Aviex CCD) distance was set to 1892 mm, covering a q-range of 0.004–0.7 Ǻ^−1^. All experiments were temperature controlled at 25 °C. Typically, 55 successive frames of 0.5 s each were recorded for both protein solution and its corresponding buffer. Each frame was first angularly averaged and the final spectrum and experimental error were obtained by averaging over all frames and subtracting the pure solvent spectrum from the sample spectrum. Intensities were scaled using the scattering of water^35^. Data analysis was based on fitting the scattering curve to an appropriate model by a least-squares method using software provided by NIST^36^ (NIST SANS Analysis version 7.0 on IGOR).

The scattering intensity of a monodispersed system of particles of identical shape can be described by the following equation^34^:


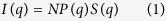


where N is the number of particles per unit volume, P(q) is the form factor revealing the specific size and shape of the scatterers and S(q) is the structure factor that accounts for the interparticle interactions. In dilute solutions, where the interactions between the objects can be neglected, S(q) is equivalent to 1.

A form factor for a simple monodispersed core-shell sphere, where the core and the shell have a uniform electron density, is given by:


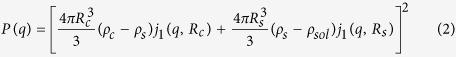



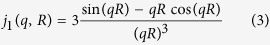


where R_c_ and R_s_ are the core and shell radii, respectively, and ρ is electron density.

### Transmission electron microscopy (TEM)

Horse spleen ferritin was obtained from Sigma. Pure ferritin-M6A protein was reconstituted with Fe^2+^ solution under slow oxidative conditions at 60 °C and pH 8.5. TEM samples were deposited on copper TEM grids (carbon film grids) by placing a 5 μl sample onto the grid for 20 s and then washing the grid with deionised water for 3 s. For samples that were stained to visualise the protein, a 5 μl addition of sodium silicotungstate was placed on the grid for 10 s after the water wash. The grid was then allowed to air dry. In between each step the grid was blotted with filter paper.

### Cell culture

Rat C6 glioma cells were cultured in DMEM medium (Gibco, Invitrogen) supplemented with 10% fetal bovine serum, 2 mM L-glutamine and penicillin-streptomycin. Puromycin (2.5 μg mL^−1^) was added to the cell culture medium to select for stably transduced cells. The cells were incubated at 37 °C in a humidified atmosphere of 95% air and 5% CO_2_.

### Construction of C6-pEIRES-HA-ferritin and C6-pEIRES-HA-ferritin-M6A cells

The murine ferritin h-chain cDNA (GenBank accession no. NM-010239) with an HA (influenza haemagglutinin) tag (HA-ferritin) and a Kozak sequence at the N-terminus was generated by RT-PCR. Additionally, a recombinant ferritin-M6A fusion protein was generated in which the M6A peptide was attached to the C-terminus of ferritin (ferritin-M6A). The two constructs were cloned into the pEIRES mammalian expression vector.

Rat C6 glioma cells were transfected with pIRESHA-ferritin/HA-ferritin-M6A plasmids stably selected with puromycin. Ferritin and ferritin-M6A expression levels were monitored by Western blot analysis.

### Western blot analysis

Cells were lysed in RIPA buffer (20 mM Tris pH 7.4, 10% glycerol (137 mM), 0.5% sodium deoxycholate, 0.1% SDS, 1% Triton X-100, 2 mM EDTA, 200 μl of 1 mM PMSF and protease inhibitor cocktail). Equal amounts of protein (30 μg/lane; Bradford method) were electrophoresed in 15% SDS polyacrylamide gel. Blocked membranes (2% BSA in 10 mM Tris-buffered saline, 0.05% Tween (TBST) for three hours at 24 °C) were incubated overnight at 4 °C with anti-HA monoclonal antibody (HA.11, 1:1000; Covance, Inc., Berkley, CA). Membranes were washed three times with TBST and incubated with goat-anti-mouse horseradish peroxidase-labelled antibody.

### Immunohistochemical and Prussian blue staining of tissue sections

Tumours were fixed in 4% paraformaldehyde for 15 min followed by thorough washes and processed for immunohistochemical and Prussian blue staining.

Hypoxyprobe-1 (anti-pimonidazole 1/50, Hypoxyprobe Gemini Kit, USA) has been used to identify hypoxic tissue areas. Sections were blocked with Dako and incubated for one hour at room temperature with biotinylated anti-mouse SP. The slides were then incubated with HRP-conjugated Streptavidin peroxidase, developed with DAB and counterstained with haematoxylin.

For iron detection, Prussian blue staining was carried out. Slides with tissue sections were incubated in the working solution by mixing equal parts of 10% potassium ferrocyanide and 20% hydrochloric acid for 40 min. Then the slides were counterstained with Fast Red. For some experiments slides were additionally treated with DAB, which enhanced the sensitivity of iron detection.

### MRI of cell suspension

For *in vitro* MRI measurements, cells were grown with or without an addition of ferric citrate (1 mM for 48 h). Iron excess was extensively washed before sample preparation. Phantom was constructed from test tubes containing various numbers of cells on top of a cooled agarose layer. MRI measurements were acquired from a coronal single slice of the phantom through the centre of the cell layer using a 9.4T Bruker MRI spectrometer. R_2_ relaxation was measured using multi-slice multi-echo pulse sequence with 30 echoes (TE = 10 ms, TR = 3 s, FOV = 4 × 4 cm, slice thickness 1 mm, matrix 256 × 256, number of averages 2). R_2_ maps were reconstructed on a pixel-wise single exponential fit of the signal intensity decay using the equation: I = I_0_e^−TE*R2^, where I is a signal intensity, I_0_ is proton density and R_2_ = 1/T_2_.

### *In vivo* MRI

All experiments on mice were carried out in accordance with the approved guidelines of the Weizmann Institute Animal care and Use Committee. Rat glioma C6 cells containing either HA-ferritin or HA-ferritin-M6A constructs were grown in DMEM and selected with 2 μg mL^−1^ of puromycin. The cells were trypsinized, washed twice with PBS and counted. For injection, cells were resuspended in PBS to the required volume with 10^6^ cells per 25 μl. 6–9 weeks old CD-1 female nude mice were inoculated subcutaneously above the gluteal muscle with two types of cells (10^6^ cells/cell type) which contained HA-ferritin and HA-ferritin-M6A constructs. Two weeks after inoculation, tumour size was monitored using MRI.

MRI measurements were acquired from a single axial slice through the centre of both tumours (HA-ferritin and HA-ferritin-M6A constructs), at 9.4T on a Bruker Biospec spectrometer with a small Quadrature coil (Transceiver 1H 100W). R_2_ relaxation was measured using multi-slice multi-echo pulse sequence with 30 echoes (TE = 8 ms, TR = 3 s, FOV = 3 × 3 cm, slice thickness 0.8 mm, matrix 256 × 256, number of averages 2). R_2_ maps were reconstructed on a pixel-wise single exponential fit of the signal intensity decay using equation: I = I_0_e^−TE*R2^, where I is a signal intensity, I_0_ is proton density and R_2_ = 1/T_2_. Three weeks after inoculation mice were sacrificed and tumours were fixed for further histology and immunohistological examination. Two mice from each group were intraperitoneally administered with pimonidazole (60 mg kg^−1^) 1 h before sacrificing.

### Tumours Mössbauer analyses

Subcutaneous tumours were generated by injecting 4 × 10^6^ single-cell suspensions of cells in 30 μl PBS into a shaved lower right flank of 7-weeks old mice. Two weeks later tumours were harvested and frozen directly in liquid nitrogen, which was followed by lyophilization. To protect the samples from room atmosphere, the samples were prepared in a glove box stored in a liquid nitrogen dewar to be transported to the Mössbauer laboratory (Le Mans France). ^57^Fe Mössbauer spectra were then collected using a conventional constant acceleration transmission spectrometer with a ^57^Co(Rh) source at 77 K by means of a bath cryostat. It is important to emphasize that the samples contain a very low content of Fe, consequently, the Mössbauer spectra exhibit extremely low statistics, despite registration times of 2–3 weeks with a 925 MBq γ-source of 57Co/Rh.

## Additional Information

**How to cite this article**: Radoul, M. *et al*. Genetic manipulation of iron biomineralization enhances MR relaxivity in a ferritin-M6A chimeric complex. *Sci. Rep.* **6**, 26550; doi: 10.1038/srep26550 (2016).

## Supplementary Material

Supplementary Information
