# Supplementary material for: Genetic manipulation of iron biomineralization enhances MR relaxivity in a ferritin-M6A chimeric complex
Source: Sci Rep. 2016 May 23;6:26550. doi: 10.1038/srep26550 (PMC4876375; doi:10.1038/srep26550)
Supplement: Supplementary Information [file srep26550-s1.pdf]

Supplementary information

Genetic manipulation of iron biomineralization enhances MR relaxivity in a ferritin-M6A chimeric complex

Marina Radoul[1†‡], Limor Lewin[2‡], Batya Cohen[1*], Roni Oren[1], Stanislav Popov[2], Geula Davidov[2], Moriel Vandsburger[1, †], Alon Harmelin[3], Ronit Bitton[4], Jean-Marc Greneche[5], Michal Neeman[1]* and Raz Zarivach [2]*.

AUTHOR ADDRESS

[1] Department of Biological Regulation, Weizmann Institute of Science, Rehovot 76100 Israel

[2] Department of Life Sciences and the National Institute for Biotechnology in the Negev, Ben-Gurion University of the Negev, POB 653, Beer-Sheva 84105, Israel

[3] Department of Veterinary Resources, Weizmann Institute of Science, Rehovot 76100 Israel

[4] Department of Chemical Engineering and Ilse Katz Institute for Nanoscale Science and Technology Ben-Gurion University of the Negev, 84105 Beer-Sheva, Israel

[5] Institut des Molécules et Matériaux du Mans (IMMM), UMR CNRS 6283 Université du Maine, Avenue Olivier Messiaen, 72085 Le Mans Cedex France

Corresponding Authors:

Michal Neeman

Department of Biological Regulation

Weizmann Institute of Science, Rehovot 76100 Israel

Email: michal.neeman@weizmann.ac.il

Phone: +972-8-9342487 Fax: +972-8-9346264

And

Raz Zarivach

Department of Life Sciences and the National Institute for Biotechnology in the Negev

Ben-Gurion University of the Negev, POB 653, Beer-Sheva 84105, Israel

Email zarivach@bgu.ac.il

Phone +972-8-64-61999 Fax +972-8-64-72970

Supplementary Table1 – diffraction data and processing statistic.

PDB code	3WNW
Protein	Ferritin-M6A
Data collection	ID14-4 - ESRF
Space group	$P4_22_12$
**Cell dimensions**	
a, b, c (Å)	218.15, 218.15, 147.69
α, β, γ (°)	90, 90, 90
Resolution (Å)	50-2.25
Rsym or Rmerge	14.3 (56.1)
I / σI	10.83 (2.59)
Completeness (%)	95.6 (95.5)
Redundancy	4.7 (3.7)
Wavelength (Å)	0.947
**Refinement**	
Resolution (Å)	2.25
No. reflections	152156
Rwork / Rfree (%)	22.04/26.53
**No. atoms**	
Protein	17176
Ligand/ion	98
Water	1374
**B-factors**	
Protein	15.36
Ligand/ion	26.39
Water	20.60
**R.m.s. deviations**	
Bond lengths (Å)	0.0196
Bond angles (°)	1.911

Data was collected from a single crystal. Values in parentheses are for the highest resolution shell.

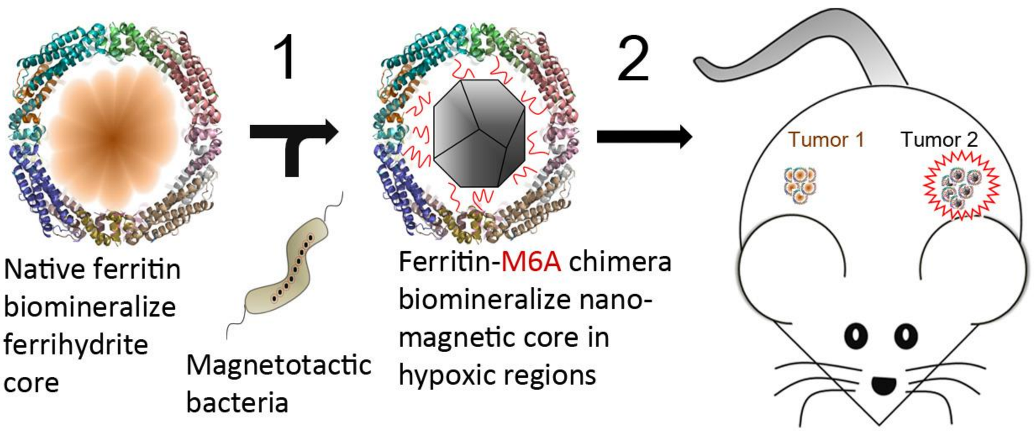

Supplementary Figure 1. C-terminal modification of mouse h-ferritin with the C-terminal tail of Mms6 (ferritin-M6A), a magnetosome-associated protein from magnetotactic bacteria (1). Such modification led to magnetic-producing ferritin *in vivo*. As a result, a tumour containing ferritin-M6A has enhanced R_2 relaxation relative to a tumour containing native ferritins under hypoxic regions (2).

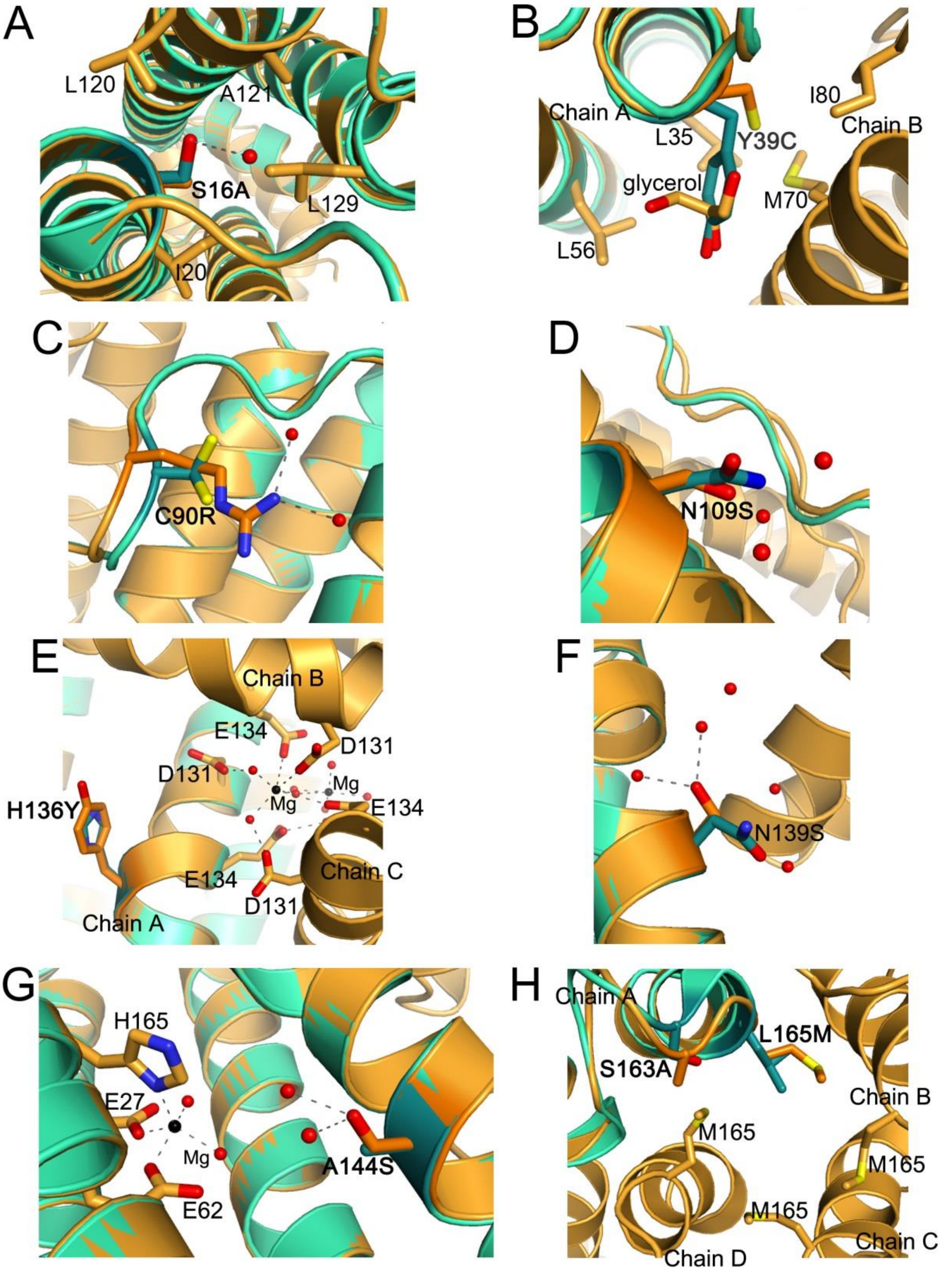

Supplementary Figure 2. Crystal structures overlap of human wild-type h-ferritin (PDB 3AJO, cyan) with mouse h-ferritin-M6A (bright orange). (A) S16A replacement leads to the loss of a hydrogen bond of Ser16 with a water molecule within the middle of the hydrophobic four-helical bundle. (B) Y39S replacement results in a void that in the mouse ferritin is filled by glycerol in the hydrophobic two-fold interface. (C) C90R replacement in the hydrophilic surface area. (D) N109S replacements of hydrophilic by other hydrophilic residue in the external surface of the ferritin. (E) H136Y replacement of a small aromatic hydrophilic residue with a larger aromatic

ring in the interior surface of the ferritin core, near the three-fold symmetry axis channel. (F) N139S replacements of hydrophilic by other hydrophilic residue in the external surface of the ferritin. (G) A144S replacements enables S144 interaction with water molecules on the inner ferritin surface, close to metal binding site A. (H) S163A replacement is located at the loop connecting helix 3 to 4, and L165M replacement which sits at the end of this loop.

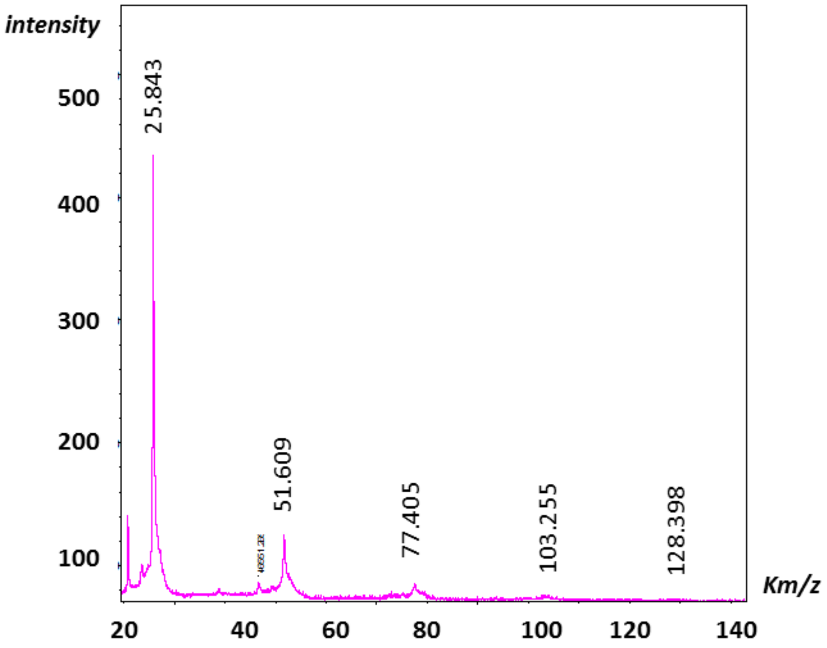

Supplementary Figure 3. MALDI-TOF analysis of purified h-ferritin-M6A. The experimentally defined size of ferritin-M6A (25.843 kDa, including the His tag at the N-terminal) is within the calculated size of modified ferritin (25.868 kDa). The other peaks represent dimers, trimmers, tetramers and pentamers resulting from a breakdown of the 24-mer assembly during the matrix ionization.

1 ul solution of purified ferritin-M6A sample (1 mg mL^{-1}) was mixed with 10 ul and 100 ul of saturated solution of sinapinic acid (MALDI matrix) and 1 ul of each mix was deposited onto the MALDI target plate. Spectral data were collected with Reflex IV (Bruker Daltonics, Germany) MALDI-TOF mass spectrometer in positive linear mode using a nitrogen laser with a wavelength of 337 nm. A total of 300 laser shots were summed for each spectrum. The mass spectrometer's parameters were optimized for the range of the m/z values from 14 to 200 kDa using Protein Calibration Standard II (Bruker Daltonics, Germany) for the calibration. Processing and analysis of the spectra were performed with Bruker FlexControl version 3.0 and FlexAnalysis version 3.0 software.

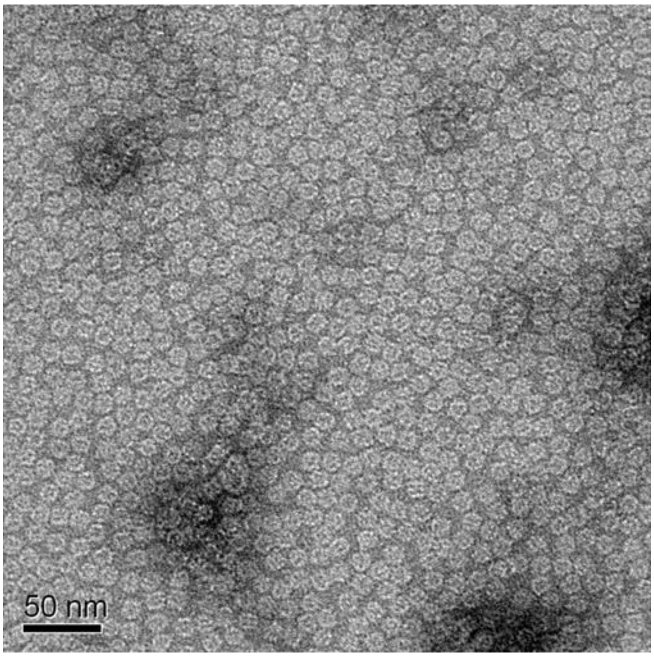

Supplementary Figure 4. TEM images of negatively stained (Uranyl Acetate) recombinant mouse h-ferritin. Black bars indicate 50 nm length.

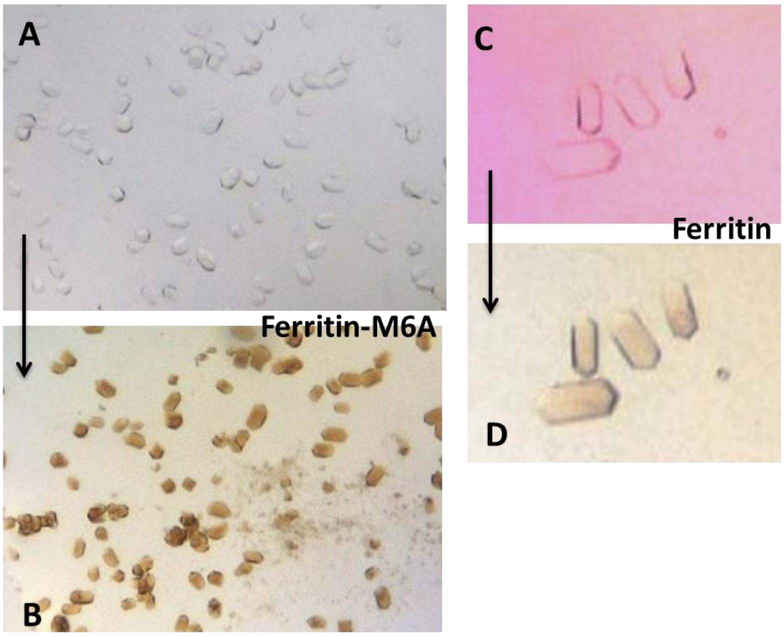

Supplementary Figure 5. Iron loading of ferritin-M6A and ferritin crystals. Crystal changed their color in response to the addition of 0.1 ul of 0.1 M ammonium ferrous sulphate into the crystallization d after crystal formation (black arrow). Colour change is the response of ferrihydrite in the ferritin core.

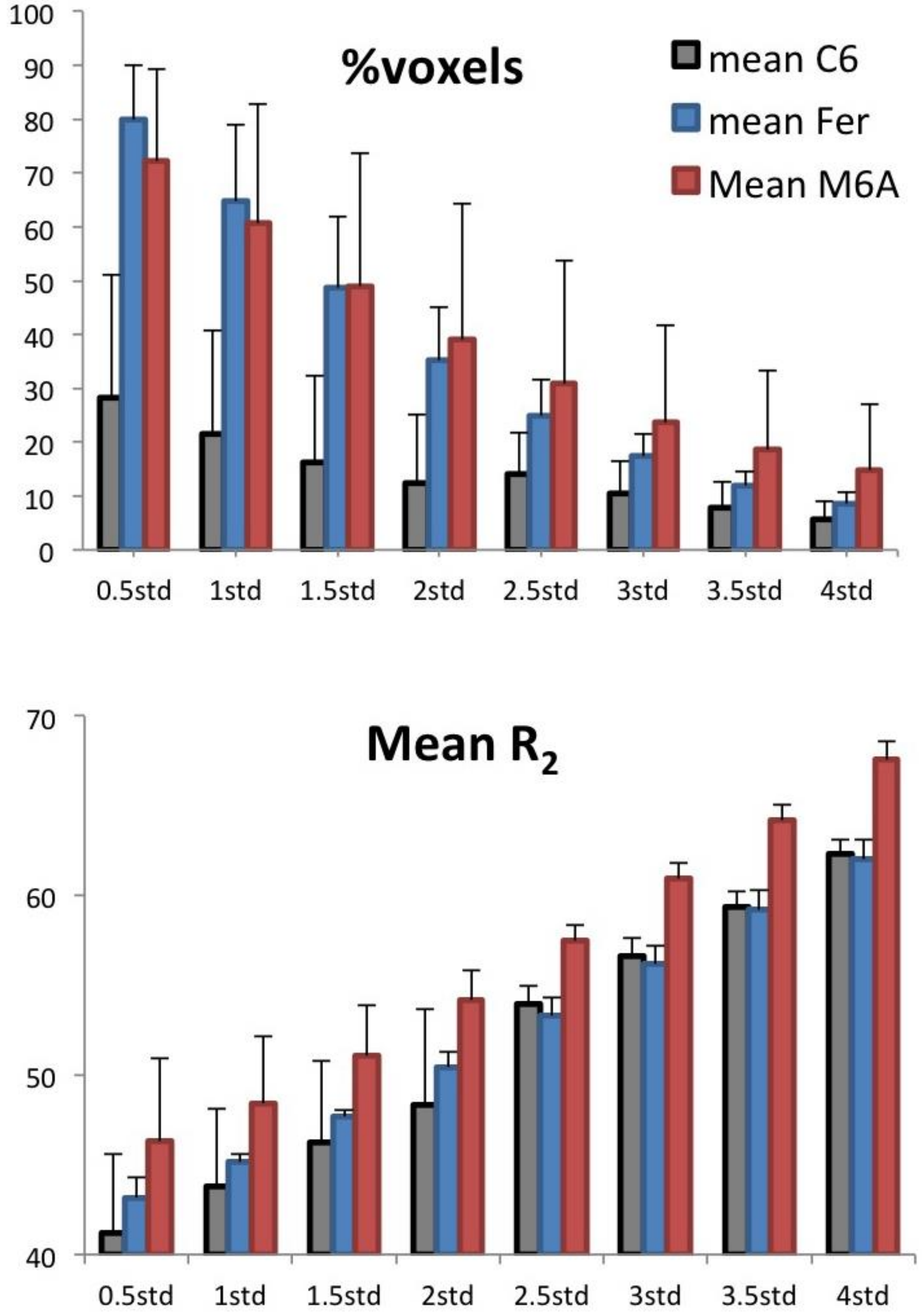

Supplementary Figure 6. Enhanced R_2 relaxation in C6 glioma subcutaneous tumour xenografts. CD-1 nude mice were inoculated subcutaneously with either wild type C6 glioma cells (n=3), C6 cells expressing HA-tagged h-ferritin (n=4) or C6 cells expressing HA-tagged h-ferritin-M6A (n=3). Analysis of the histograms of R_2 relaxation maps showed elevated relaxation for both ferritin and ferritin-M6A

overexpressing tumours. Higher relaxation rates and elevated fractions of voxels with high relaxation rates (>2.5 STD) were observed for the ferritin-M6A tumours relative to the wild type or ferritin-overexpressing tumours.

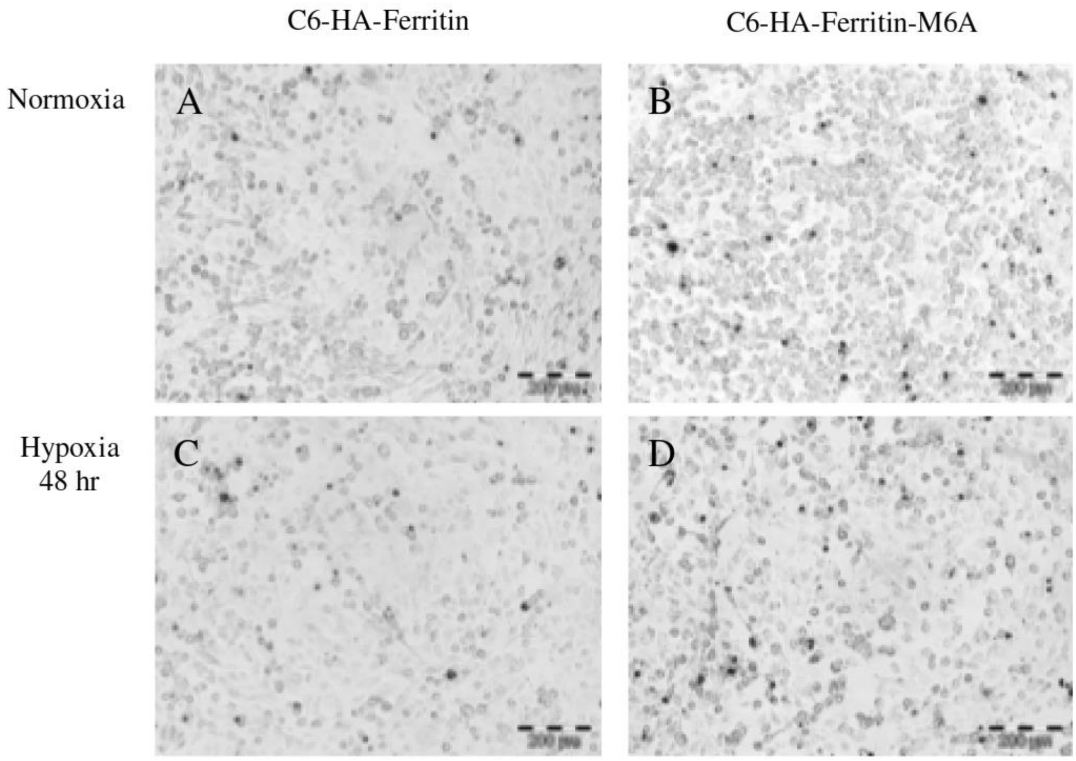

Supplementary Figure 7. Hypoxia-regulated iron biomineralization for cells expressing ferritin-M6A. C6 rat glioma cells overexpressing either HA-tagged ferritin of HA-tagged ferritin-M6A were cultured at normoxic and hypoxic conditions. Ferritin-M6A-expressing cells exhibited higher iron content both under normoxic (5% CO_2, 95% air, 37 °C) and hypoxic (5% CO_2, 1% O_2, 94% N_2, 37°C) conditions. The cells were incubated with 4% formalin in PBS and stained with DAB-enhanced Prussian blue staining.

In vitro hypoxia test

Rat glioma C6-HA-ferritin and C6-HA-ferritin-M6A cells (10^5 cells/well) were grown on untreated six-well plates with or without ferric citrate (1 mM) supplementation. Cells were grown under either normoxic or hypoxic conditions (1 % O_2) for varying periods of time (48 h, 72 h and 96 h). After incubation, cells were washed twice with PBS and fixed with 3 % paraformaldehyde prepared in PBS.

Prussian blue staining on cells

The cells were incubated in the working solution (a mix of equal parts of 10 % potassium ferrocyanide and 20 % hydrochloric acid) for 40 min. Duplicate wells were additionally treated with DAB for enhancement of the sensitivity of iron detection.
